# Protocadherin-αC2 is required for diffuse projections of serotonergic axons

**DOI:** 10.1038/s41598-017-16120-y

**Published:** 2017-11-21

**Authors:** Shota Katori, Yukiko Noguchi-Katori, Atsushi Okayama, Yoshimi Kawamura, Wenshu Luo, Kenji Sakimura, Takahiro Hirabayashi, Takuji Iwasato, Takeshi Yagi

**Affiliations:** 1 KOKORO-Biology, Laboratories for Integrated Biology, Graduate School of Frontier Biosciences, Osaka University, 1-3 Yamadaoka, Suita, Osaka 565-0871 Japan; 2 Division of Neurogenetics, National Institute of Genetics, 1111 Yata, Mishima, Shizuoka 411-8540 Japan; 3 Department of Physiology, Keio University School of Medicine, 35 Shinanomachi, Sinjuku, Tokyo 113-0021 Japan; 4 Department of Genetics, SOKENDAI (The Graduate University for Advanced Studies), Mishima, Shizuoka 411-8540 Japan; 5 Department of Cellular Neurobiology, Brain Research Institute, Niigata University, 1-757 Asahimachidoori, Chuoku, Niigata 951-8585 Japan; 6 AMED-CREST, Tokyo, 100-0004 Japan

## Abstract

Serotonergic axons extend diffuse projections throughout various brain areas, and serotonergic system disruption causes neuropsychiatric diseases. Loss of the cytoplasmic region of protocadherin-α (Pcdh-α) family proteins, products of the diverse clustered *Pcdh* genes, causes unbalanced distributions (densification and sparsification) of serotonergic axons in various target regions. However, which *Pcdh-α* member(s) are responsible for the phenotype is unknown. Here we demonstrated that *Pcdh-αC2* (*αC2*), a *Pcdh-α* isoform, was highly expressed in serotonergic neurons, and was required for normal diffusion in single-axon-level analyses of serotonergic axons. The loss of αC2 from serotonergic neurons, but not from their target brain regions, led to unbalanced distributions of serotonergic axons. Our results suggest that αC2 expressed in serotonergic neurons is required for serotonergic axon diffusion in various brain areas. The αC2 extracellular domain displays homophilic binding activity, suggesting that its homophilic interaction between serotonergic axons regulates axonal density via αC2′s cytoplasmic domain.

## Introduction

Serotonergic somata are located in the midline of the brainstem, and serotonergic neurons project their axons throughout the entire brain and spinal cord^1–3^. The serotonergic system regulates a wide range of brain functions, including circadian rhythm, thermoregulation, response to stress and pain, and feeding, maternal, aggressive and sexual behaviors^4^.

The ontogeny of the serotonergic projections involves three main steps: the initial orientation of axons along the anterior-posterior axis, the guidance of axons along their main pathways, and the terminal projections to the targets of innervation^3,5^. Many of the molecular mechanisms underlying these steps have been identified. For example, growth associated protein 43 (GAP-43) and a microtubule-associated protein, stable tubule only polypeptide (STOP) influence the growth of serotonergic axons^6,7^, and protocadherin-α (Pcdh-α) regulates the distribution of serotonergic axons in many target brain areas^8^.

The *Pcdh-α* genes belong to the clustered Pcdh families, which also include the *Pcdh-β* and *Pcdh-γ* genes^9,10^. All of the *Pcdh-α* genes encode type I transmembrane proteins that have the following distinct domains: six extracellular cadherin domains, a transmembrane domain, and a cytoplasmic domain. The *Pcdh-α* mRNA consists of a variable exon, encoding extracellular, transmembrane, and juxtamembrane cytoplasmic domains, and three (or four) constant exons, encoding the common cytoplasmic tail^9,10^. Mutants lacking the common C-terminal cytoplasmic tail exhibit learning defects^11^ and aberrantly distributed serotonergic axons^8^. These findings indicate that the common cytoplasmic tail is essential for the normal distribution of serotonergic projections in the target brain regions. In addition, each extracellular domain of Pcdh-α family proteins possesses homophilic binding activity in cell-to-cell interaction^12^. A diversity of Pcdh-γ proteins is used for the self-repulsion of dendrites, based on isoform-specific homophilic interaction^13^. However, the role of the divergent Pcdh-α proteins in normal serotonergic projections remains unknown. In the present study, we found that *Pcdh-αC2* (*αC2*), one *Pcdh-α* isoform, is predominantly expressed by serotonergic neurons among *Pcdh-α* isoforms, and essential for preventing their axon from being too dense or too sparse and for promoting the diffuse projections of serotonergic axons. Here we propose that serotonergic axons predominantly use αC2 among the Pcdh-α members for appropriate axonal projection.

## Results

### Deletion of the variable exons α11 to αC2 induces abnormal serotonergic projections, while deletion of α2 to α11 does not

**Figure 1:**
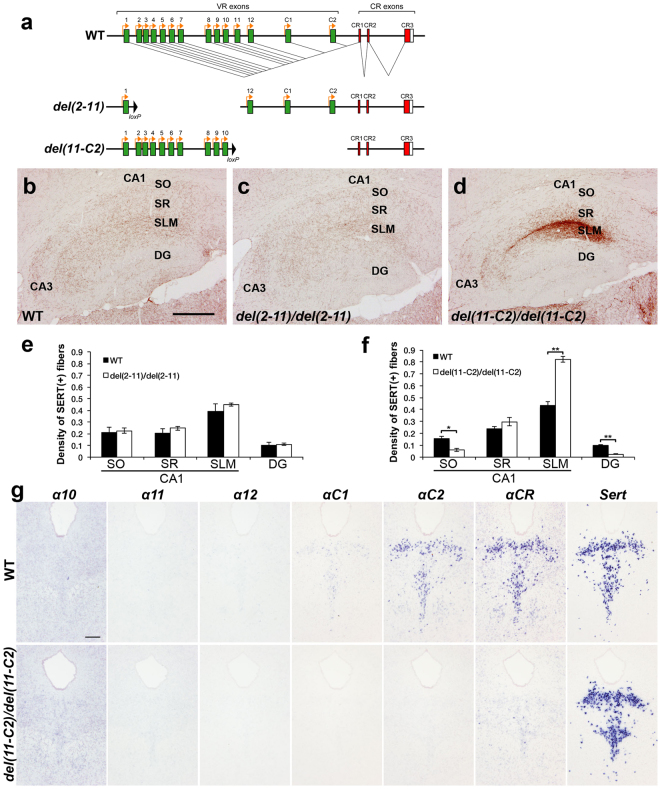
*Pcdha* ^*del*(*11-C2*)*/del*(*11-C2*)^ mice, but not *Pcdha* ^*del*(*2-11*)*/del*(*2-11*)^ mice, exhibit altered distributions of serotonergic axons. (**a**) Wild-type (WT) *Pcdh-α* genes consist of exons (*1–12*, *C*1 and *C2*; green) in a variable region (VR) and exons (*CR1–CR3*; red) in a constant region (CR). The individual variable exons are transcribed from their own promoters. A *Pcdh-α* transcript is produced from one variable exon and three or four constant exons by splicing. In the *del*(*2-11*) allele, exons *α2–α11* were deleted. In the *del*(*11-C2*) allele, exons *α11*, *α12*, *αC1*, and *αC2* were deleted. (**b**–**d**) Serotonergic axons in WT, *Pcdha* ^*del*(*2-11*)*/del*(*2-11*)^, and *Pcdha* ^*del*(*11-C2*)*/del*(*11-C2*)^ mice were detected by an anti-serotonin transporter (SERT) antibody. The distribution of serotonergic axons in *Pcdha* ^*del*(*2-11*)*/del*(*2-11*)^ mice (**c**) was similar to that in WT mice (**b**), whereas the serotonergic fibers of *Pcdha* ^*del*(*11-C2*)*/del*(*11-C2*)^ mice (**d**) were densified in the stratum lacnosum-moleculare (SLM) of CA1, and sparsified in the stratum oriens (SO) of CA1 and in the dentate gyrus (DG), compared with WT mice. SR, stratum radiatum. Scale bar: (**b**–**d**), 500 µm. (**e**) Quantification of SERT(+) fibers in WT (*n* = 6) and *Pcdha* ^*del*(*2-11*)*/del*(*2-11*)^ mice (*n* = 5). (**f**) Quantification of SERT(+) fibers in WT (*n* = 6) and *Pcdha* ^*del*(*11-C2*)*/del*(*11-C2*)^ mice (*n* = 5). *p < 0.05, **p < 0.01. Mean ± SEM. (**g**) Expression analysis by *in situ* hybridization using probes for *α10*, *α11*, *α12*, *αC1*, *αC2*, *αCR*, and *Sert* in adjacent coronal sections of the dorsal raphe nucleus of WT and *Pcdha* ^*del*(*11-C2*)*/del*(*11-C2*)^ mice.

We previously reported that mice in which the *Pcdh-α* constant region (*CR*) exon was deleted (*Pcdha* ^*∆CR/∆CR*^) and those lacking the Pcdh-α cytoplasmic tail (*Pcdha* ^*∆A/∆A*^) exhibit abnormal serotonergic projections^8^. Although these results indicated that *Pcdh-α* genes are essential for normal serotonergic projections, whether all of the Pcdh-α members or just specific ones are necessary was still unclear. In the current study, we analyzed two different *Pcdh-α* variable exon-deletion mutant lines: *Pcdha* ^*del*(*2-11*)*/del*(*2-11*)^ mice, in which the variable exons *α2* to *α11* were deleted, and *Pcdha* ^*del*(*11-C2*)*/del*(*11-C2*)^ mice, in which the variable exons *α11* to *αC2* were deleted^14^ (Fig. 1a). In the *Pcdha* ^*del*(*2-11*)*/del*(*2-11*)^ mice, the distribution of serotonergic [serotonin transporter (SERT)-immunopositive] axons in the hippocampus was similar to that of wild-type (WT) mice (Fig. 1b,c). Quantitative analysis of the SERT(+) axon density revealed no significant difference in the density of serotonergic axons in each layer in CA1 and the dentate gyrus (DG) between *Pcdha* ^*del*(*2-11*)*/del*(*2-11*)^ and WT mice (Fig. 1e). In contrast, the *Pcdha* ^*del*(*11-C2*)*/del*(*11-C2*)^ mice showed marked densification of serotonergic axons in the stratum lacunosum-moleculare (SLM) of CA1 (Fig. 1b,d). Quantitative analysis of the SERT(+) axon density revealed that in the *Pcdha* ^*del*(*11-C2*)*/del*(*11-C2*)^ mice, the density was significantly higher in the SLM of CA1 and lower in the stratum oriens (SO) of CA1 and the DG, compared with WT mice (Fig. 1f). These results indicated that the genomic region from the variable exons *α*12 to *αC2* is indispensable for the formation of normal serotonergic projections, whereas the genomic region from the variable exons *α2* to *α11* is dispensable. Next, we examined the expression level of each *Pcdh-α* gene by *in situ* hybridization. In the raphe nuclei of WT mice, the *αC2* transcript was strongly expressed, and the *αC1* transcript was weakly expressed; in contrast, transcripts for the *α*10 to *α12* genes were not detected (Fig. 1g). These findings suggested that the *αC2* transcript is the dominant *Pcdh-α* transcript in the serotonergic neurons of the raphe nuclei.

We previously reported that in *Pcdha* ^*del*(*11-C2*)*/del*(*11-C2*)^ mice, stochastic and weak expression patterns of *α10* are severely altered to *αC2*-like constitutive and strong expression patterns in several brain regions including the cerebral cortex, hippocampus, and cerebellar Purkinje cells, and that the total expression levels of all of the *Pcdh-α* isoforms is maintained^14^. In contrast, in the raphe nuclei of these mice, the expression level of total *Pcdh-α* transcripts, which was detected by the common *Pcdh-α CR* exon probe, was markedly lower, and the expression level of *α10* was grossly normal (Fig. 1g). These results indicated that the regulation of *Pcdh-α* gene expression in the raphe nuclei is different from that in the cerebral cortex, hippocampus, and cerebellum.

### αC2 is essential for normal serotonergic projections

**Figure 2:**
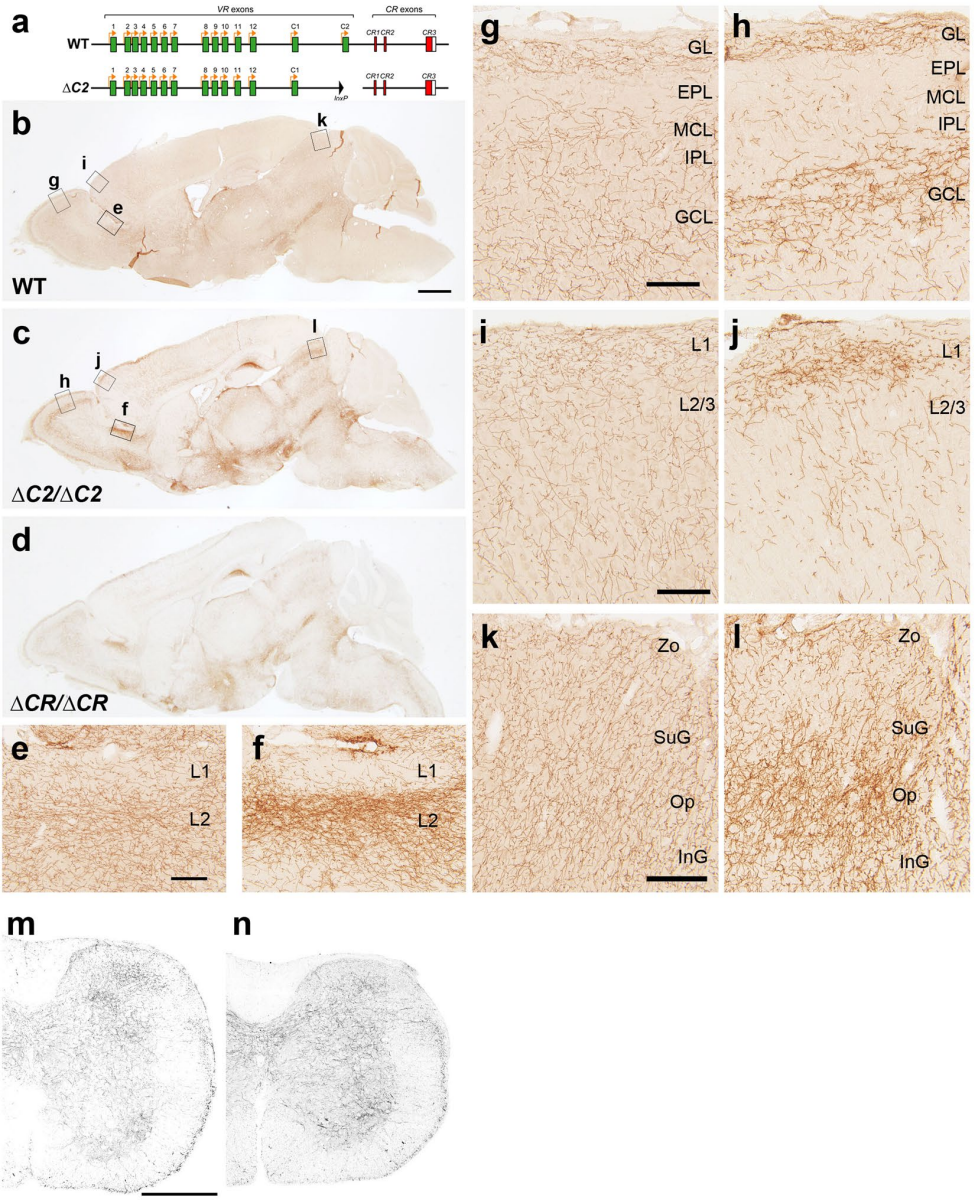
*Pcdha* ^*∆C2/∆C2*^ mice exhibit altered distributions of serotonergic axons. (**a**) WT and *∆C2* alleles. In the *∆C2* allele, the *αC2* exon was deleted. (**b**–**d**) Parasagittal sections of WT (**b**), *Pcdha* ^*∆C2/∆C2*^ (**c**) and *Pcdha* ^*∆CR/∆CR*^ brains (**d**) were stained with an anti-SERT antibody. Boxed areas (**e**–**l**) in (**b**) and (**c**) were magnified in the following panels. (**e**,**f**) In layer 2 (L2) of the anterior olfactory nucleus, the density of SERT(+) axons in *Pcdha* ^*∆C2/∆C2*^ mice (**f**) was higher than that in WT mice (**e**). (**g**,**h**) In the granule cell layer (GCL) of the olfactory bulb, SERT(+) axons of *Pcdha* ^*∆C2/∆C2*^ mice (**h**) showed densification, compared with that of WT (**g**). EPL, external plexiform layer; GL, glomerular layer; IPL, inner plexiform layer; MCL, mitral cell layer. (**i**,**j**) In L1 of the somatomotor cortex, SERT(+) axons of *Pcdha* ^*∆C2/∆C2*^ mice (**j**) showed densification, compared with that of WT (**i**). (**k**, **l**) In the optic nerve layer (Op) of the superior colliculus, the density of SERT(+) axons of *Pcdha* ^*∆C2/∆C2*^ mice (**l**) was higher, compared with that of WT mice (**k**). InG, intermediate gray layer; SuG, superficial gray layer; Zo, zonal layer. (**m**,**n**) SERT(+) axons in the spinal cord of *Pcdha* ^+*/∆C2*^ (**m**) and *Pcdha* ^*∆C2/∆C2*^ mice (**n**). Scale bars: 1 mm (**b**–**d**); 100 µm (**e**–**l**); 200 µm (**m**,**n**).

**Figure 3:**
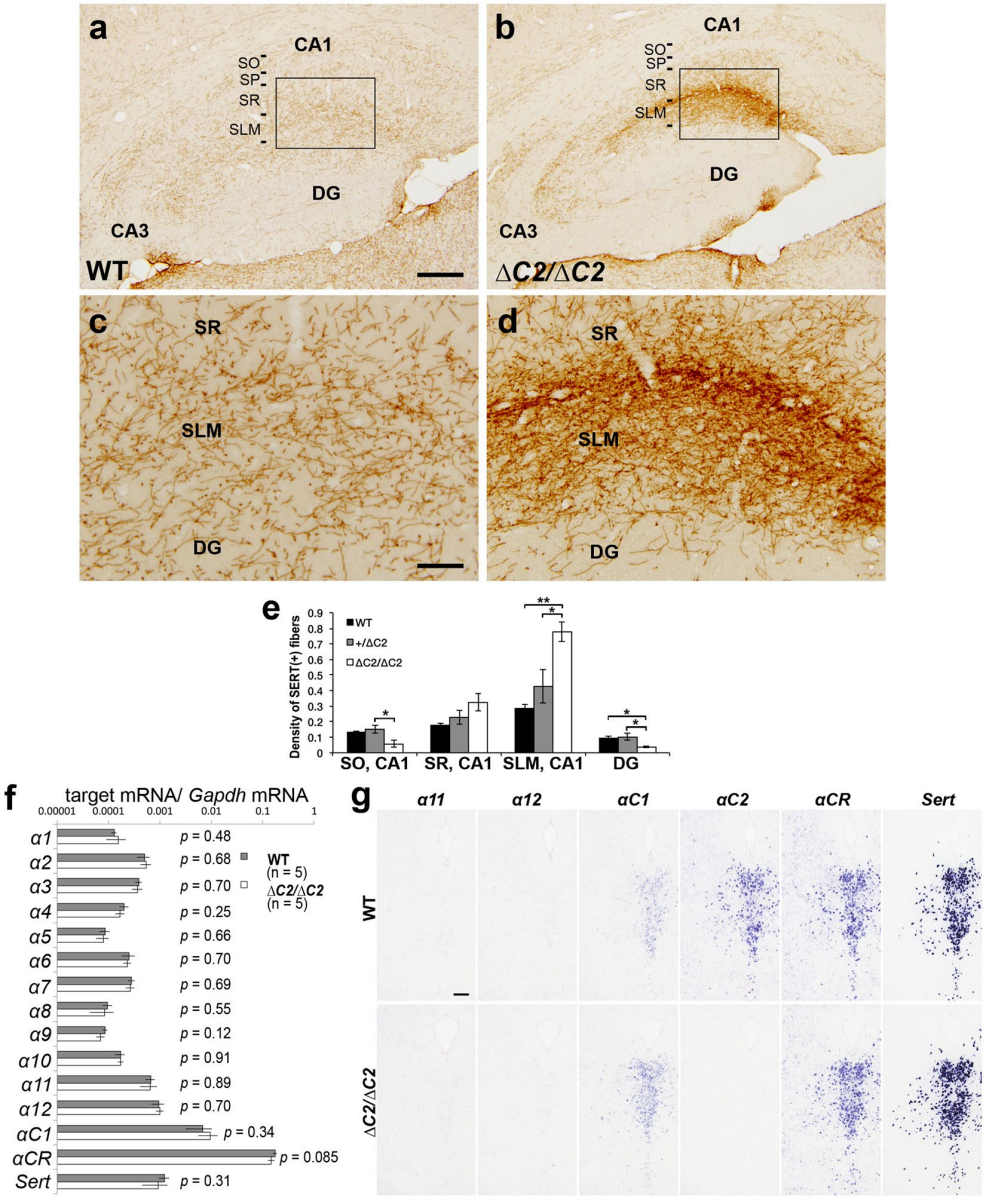
*α*C2 protein is required for normal serotonergic projections. (**a–d**) Serotonergic axons in the hippocampus of WT (**a**,**c**) and *Pcdha* ^*∆C2/∆C2*^ (**b**,**d**) mice were detected with an anti-SERT antibody. Panels (c) and (d) are higher-magnification images of the boxed areas in (**a**) and (**b**), respectively. SP, stratum pyramidale. (**e**) Quantification of SERT(+) fibers in the hippocampus of WT (*n* = 4), *Pcdha* ^+*/∆C2*^ (*n* = 3), and *Pcdha* ^*∆C2/∆C2*^ (*n* = 4) mice. *p < 0.05, **p < 0.01. Mean ± SEM. (**f**) Quantitative RT-PCR for the *Pcdha* and *Sert* genes. There was no significant difference between WT and *Pcdha* ^*∆C2/∆C2*^ KO mice. Mean ± SEM. (**g**) Expression analysis by *in situ* hybridization using probes for *α11*, *α12*, *αC1*, *αC2*, *αCR*, and *Sert* in adjacent coronal sections of the dorsal raphe nucleus of WT and *Pcdha* ^*∆C2/∆CR*^ mice. Scale bars: (**a**,**b**), 200 µm; (**c**,**d**) 50 µm; 200 μm (**g**).

Given that the *Pcdha* ^*del*(*11-C2*)*/del*(*11-C2*)^ mice exhibited abnormal serotonergic projections and *αC2* was strongly expressed in the raphe nuclei, we speculated that *αC2* was essential for normal serotonergic projections. To test this possibility, we generated *∆C2* alleles (Fig. 2a). α*C2* KO (*Pcdha* ^*∆C2/∆C2*^) mice showed abnormal serotonergic projections in many target brain areas, compared with control mice (Fig. 2b,c). In control mice, serotonergic axons extended diffuse projections throughout various brain areas, including the anterior olfactory nucleus, the olfactory bulb, the cortex, and the superior colliculus (Fig. 2b,e,g,i,k). In contrast, in these regions, *αC2* KO mice exhibited both the densification and sparsification of serotonergic axons (Fig. 2c,f,h,j,l). In the spinal cord, the serotonergic axon density in *αC2* KO mice was grossly normal, compared with control mice (Fig. 2m,n). In the hippocampus, the density of serotonergic axons in *αC2* KO mice was significantly higher in the SLM of CA1, and lower in the SO of CA1 and the DG, compared with WT mice (Fig. 3a–e). We quantified the transcripts of *Sert* and other Pcdh-α members in whole brains by quantitative reverse transcription (qRT)-PCR, and observed no significant differences in their expression levels between the WT and *αC2* KO mice (Fig. 3f). *In situ* hybridization analysis revealed that the expression levels of *α11*, *α12*, *αC1*, and *αCR* in *αC2* KO mice were similar to those in WT mice in the raphe nuclei (Fig. 3g). In the raphe nuclei of the *αC2* KO mice, the expression of *αC2* was completely lost, although the expression of *αC1* remained (Fig. 3f). These results indicated that *αC2* expression is essential for the normal diffusion of serotonergic projections in the target brain regions.

### αC2 expression in serotonergic neurons is essential for normal serotonergic projections

**Figure 4:**
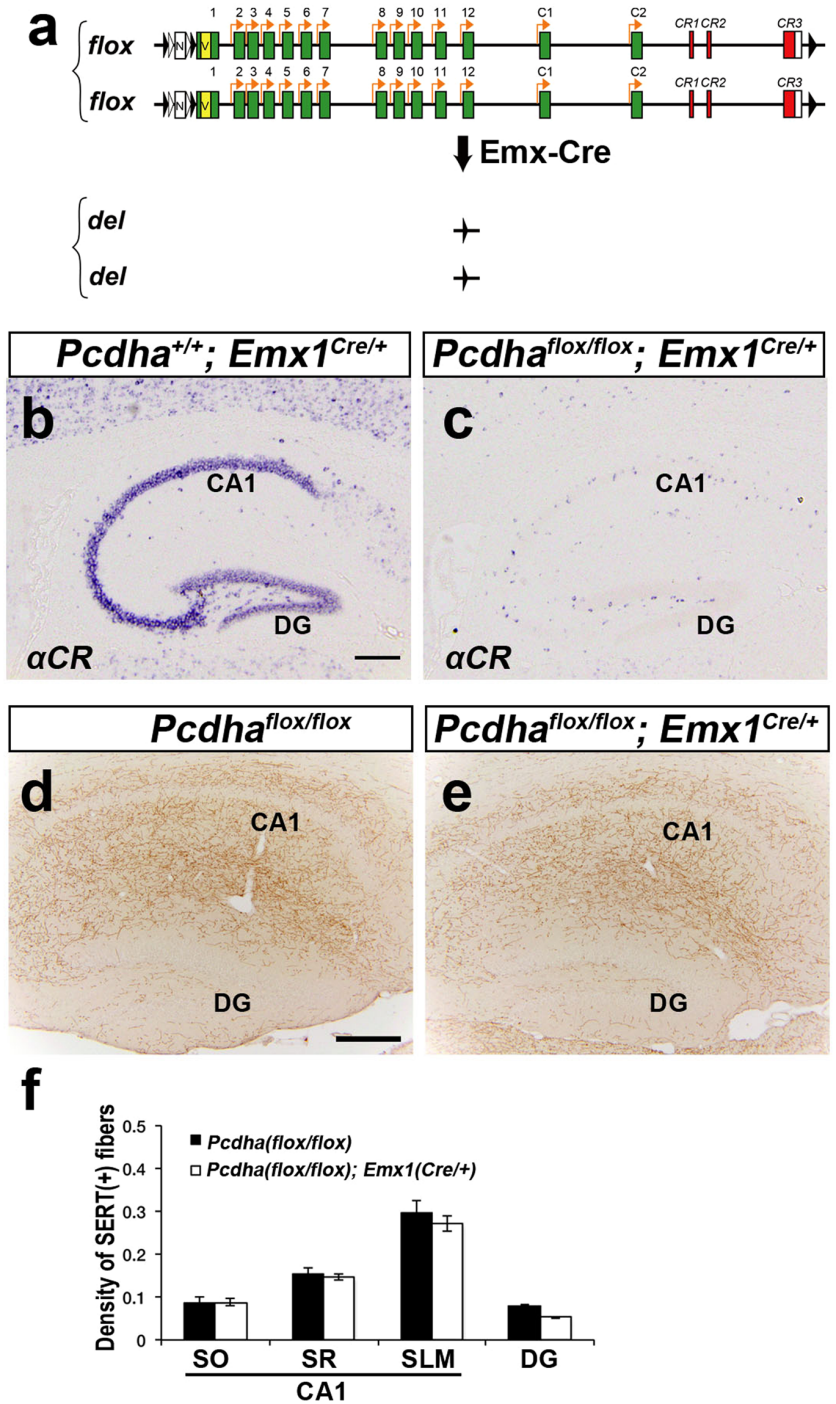
Dorsal telencephalon-specific *Pcdh-α* KO mice exhibit normal serotonergic projections. (**a**) In the *flox* allele, *loxP-FRT-neo* ^*r*^ *-FRT-loxP-Myc-Venus* and *loxP* sequences were inserted upstream of the *α1* exon and downstream of the *CR3* exon, respectively. Telencephalon-specific *Pcdh-α* KO mice were homozygous for the *Pcdhα flox* allele in *Emx1* ^*Cre/*+^. (**b**,**c**) *In situ* hybridization analysis with and *αCR* probe in the hippocampus. The expression level of the total *Pcdh-α* mRNA was markedly decreased in the hippocampus of *Pcdha* ^*flox/flox*^ *; Emx1* ^*Cre/*+^ mice (**c**), compared with controls (*Pcdha* ^+*/*+^ *; Emx1* ^*Cre/*+^ *mice*) (**b**). (**d**,**e**) In the hippocampus, the SERT(+) axonal distributions in the *Pcdha* ^*flox/flox*^ *; Emx1* ^*Cre/*+^ mice (**e**) were normal compared with controls (*Pcdha* ^*flox/flox*^, **d**). (**f**) Quantification of SERT(+) fibers in the hippocampus of control *Pcdha* ^*flox/flox*^ (*n* = 3) and *Pcdha* ^*flox/flox*^ *; Emx1-Cre* mice (*n* = 3). The density of SERT(+) axons in the hippocampus showed no significant difference. Scale bars, 200 μm (**b**–**e**).

*Pcdh-α* genes are expressed not only in serotonergic neurons, but also in other types of neurons in almost all brain regions, including the hippocampus^8^. Therefore, it was not clear whether the Pcdh-α expressed by serotonergic neurons or by other neurons in their target brain regions controls the serotonergic innervation. To address this question, we generated brain region-specific *Pcdh-α* deficient mice using the Cre-loxP system. In the *Pcdha flox* allele, *loxP* sequences were inserted upstream and downstream of the *Pcdh-α* gene cluster (Fig. 4a).

In dorsal telencephalon-specific *Pcdh-α* KO mice (*Pcdha* ^*flox/flox*^ *; Emx1* ^+*/Cre*^), *Pcdh-α* gene expression was lost in almost all of the cells in the hippocampus and cerebral cortex (Fig. 4b,c). In the dorsal telencephalon of *Emx1* ^+*/Cre*^ mice, Cre recombinase is expressed in excitatory neurons but not in inhibitory neurons^15^. Thus, almost all of the cells expressing *Pcdh-α* in the hippocampus of the dorsal telencephalon-specific *Pcdh-α* KO mice were thought to be inhibitory neurons. The serotonergic projections in these mice were grossly normal in the hippocampus, compared with control mice (*Pcdha* ^*flox/flox*^) (Fig. 4d–f). This result indicated that the expression of *Pcdh-α* genes in hippocampal excitatory neurons is dispensable for normal serotonergic projection.

**Figure 5:**
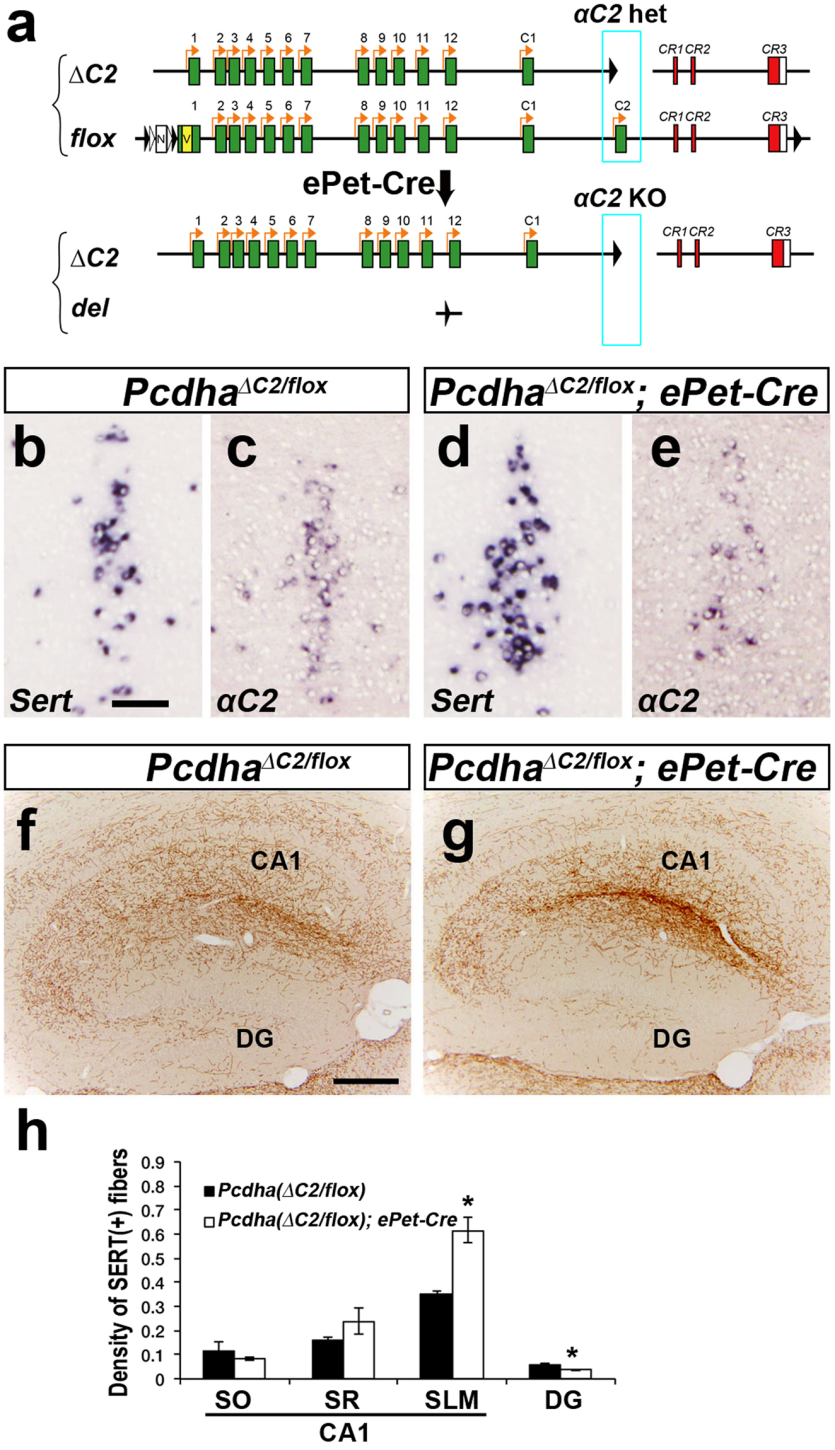
Serotonergic neuron-specific *αC2* KO mice exhibit abnormal serotonergic projections. (**a**) Serotonergic-neuron-specific *αC2* KO mice had heterozygous *Pcdhα flox* and *∆C2* alleles with the *ePet-Cre* transgene. (**b–e**) *In situ* hybridization analysis with Serotonin transporter (*Sert*) and *αC2* probes in the median raphe nuclei using adjacent sections. Almost all the *Sert*-positive neurons were *αC2-*positive in the *Pcdha* ^*flox/∆C2*^ control (**c**); in contrast, the *αC2-*positive neurons were decreased among the *Sert*-positive neurons of the *Pcdha* ^*flox/∆C2*^ *; ePet-Cre* mice (**e**). (**f**,**g**) *Pcdha* ^*flox/∆C2*^ *; ePet-Cre* mice exhibited abnormal SERT(+) axonal distributions in the hippocampus (**g**) compared with controls (*Pcdha* ^*flox/∆C2*^) (**f**). (**h**) Quantification of SERT(+) fibers in the hippocampus of control *Pcdha* ^*flox/∆C2*^ (*n* = 3) and *Pcdha* ^*flox/∆C2*^ *; ePet-Cre* mice (*n* = 3). *p < 0.05, Mean ± SEM. Scale bars: 100 μm (**b**–**e**); 200 μm (**f**,**g**).

Next, we generated serotonergic neuron-specific *αC2* KO mice (*Pcdha* ^*flox/∆C2*^ *; ePet-Cre*) using the ePet-Cre line^1^ (Fig. 5a). Serotonergic neuron-specific *αC2* KO mice (*Pcdha* ^*flox/∆C2*^ *; ePet-Cre*) showed partial deficiency of *αC2* expression in *Sert*-positive serotonergic neurons [50% of the *Sert*(+) cells in the median raphe nucleus were *αC2*(+):23 *αC2*(+) cells in 47 *Sert*(+) cells], and displayed abnormal serotonergic projections in the hippocampus, compared with control mice (*Pcdha* ^*flox/∆C2*^) (Fig. 5b–g). The density of SERT(+) axons in the serotonergic neuron-specific *αC2* KO mice was significantly higher in the SLM of CA1 and lower in the DG, compared with WT mice (Fig. 5h). The serotonergic neuron-specific *αC2* KO mice are not simple, because not only biallelic *αC2* exons but also hemi-allelic exons from *α1* to *αC1* are also deleted in the serotonergic neurons. We previously reported that *Pcdha* ^+*/∆CR*^ mice, which at least lack the expression of *α1* to *αC1* from the hemi-allele, show normal serotonergic projection in the hippocampus^8^. These results indicated that the loss of biallelic *αC2* expression in serotonergic neurons leads to aberrant serotonergic projection in the hippocampus.

### αC2 protein is required for serotonergic axon diffusion in the hippocampus

**Figure 6:**
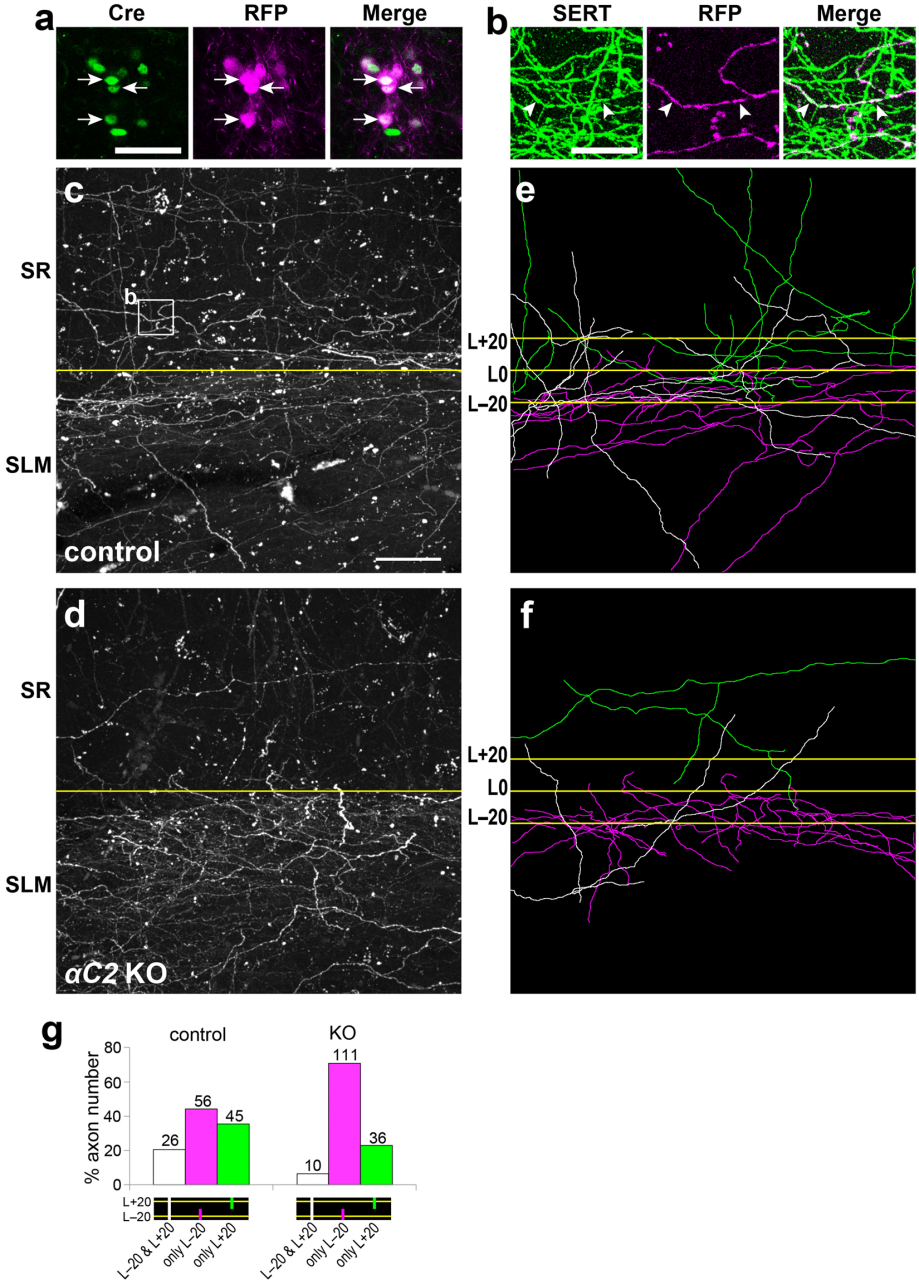
Diffusion of serotonergic axons is suppressed in the hippocampus of *αC2* KO mice. (**a**–**f**) Viral vector (AAV-*EF1α-DIO-tRFP-WPRE*) was transferred into serotonergic neurons in the raphe nuclei of *Sert-Cre* mice. (**a**) Coronal sections were immuno-stained with an anti-Cre antibody, and RFP (magenta) was expressed in Cre(+) cells (green) in the median raphe nucleus (arrows) of *Sert-Cre* mice. (**b**) A portion of SERT(+) axons (green) was RFP-positive (magenta, arrowheads). (**c**,**d**) RFP(+) axons in control (*Sert-Cre*) (**c**) and *αC2* KO (*Pcdha* ^*∆C2/∆C2*^; *Sert-Cre*) mice (**d**) were detected in sagittal sections. (**e**,**f**) The RFP(+) axons crossing the 20-μm inner line (L − 20) and/or the 20-μm outer line (L + 20) from the SLM/SR boundary (L0) were traced in control (**e**) and *αC2* KO mice (**f**). Panels (e) and (f) were depicted from panels (c) and (d), respectively. White, magenta, or green lines indicate axons crossing both lines, only L − 20, or only L + 20, respectively (**e**,**f**). (**g**) RFP(+) axons crossing L − 20 and/or L + 20 were counted in control (n = 4 sections, 3 mice) and *αC2* KO mice (n = 3 sections, 2 mice). Scale bars: 50 μm (**a**); 10 μm (**b**); 40 μm (**c**–**f**).

The loss of αC2 causes serotonergic axon densification in the SLM of the hippocampus. To elucidate how the axonal densification occurs morphologically, we sparsely labeled serotonergic axons by injecting an adeno-associated virus vector (AAV-*EF1α-DIO-tRFP-WPRE*), in which Cre expression induces red fluorescent protein (RFP) expression, into the raphe nuclei of *Sert-Cre* mice^16^. Cre(+) cells in the raphe nuclei specifically expressed RFP (Fig. 6a). A portion of serotonergic axons was labeled by RFP in both control (*Sert-Cre*) and *αC2* KO (*Pcdha* ^*∆C2/∆C2*^; *Sert-Cre*) mice (Fig. 6b–d). We did not find any axons that were in continuous contact with each other, forming thick bundles with multiple axons, or clumps with other single axons in either the control (n = 5 sections, 2 mice) or *αC2* KO mice (n = 5 sections, 4 mice). To analyze these axonal routes, we traced the RFP(+) axons passing through a 20-µm inner line (L − 20) in the SLM or a 20-µm outer line (L + 20) in the stratum radiatum (SR) from the boundary between the SLM and SR (L0) (Fig. 6c–f). The ratio of RFP(+) axons crossing L − 20 to those in L + 20 in the *αC2* KO mice was higher than that in control mice [control, 82 axons at L − 20, and 71 axons at L + 20 (3 sections from 2 mice); KO, 121 axons at L − 20, and 46 axons at L + 20, Fisher’s exact test, *p* = 0.0005, Fig. 6g]. This finding was consistent with the result of the SERT-immunopositive intensity experiment (Fig. 3a–e). Next, we analyzed the RFP(+) axons crossing both L − 20 and L + 20. The probability that RFP(+) axons passing through L − 20 also passed through L + 20 in the *αC2* KO mice was significantly lower than that in control mice [control, 26 of 82 axons (3 sections from 2 mice); KO, 10 of 121 axons (3 sections from 4 mice), Fisher’s exact test, *p* < 0.0001, Fig. 6g]. The probability that RFP(+) axons passing through L + 20 also passed through L − 20 in the *αC2* KO mice was also significantly lower than that in control mice [control, 26 of 71 axons (3 sections from 2 mice); KO, 10 of 46 axons (3 sections from 4 mice), Fisher’s exact test, *p* = 0.038, Fig. 6g]. These results indicated that the serotonergic axons in *αC2* KO mice were more suppressed from crossing the SLM/SR boundary than those in control mice. These results suggested that αC2 in the serotonergic axons is required for their axonal diffusion.

### αC2 protein is required for the outward diffusion of serotonergic axons in the olfactory bulb

**Figure 7:**
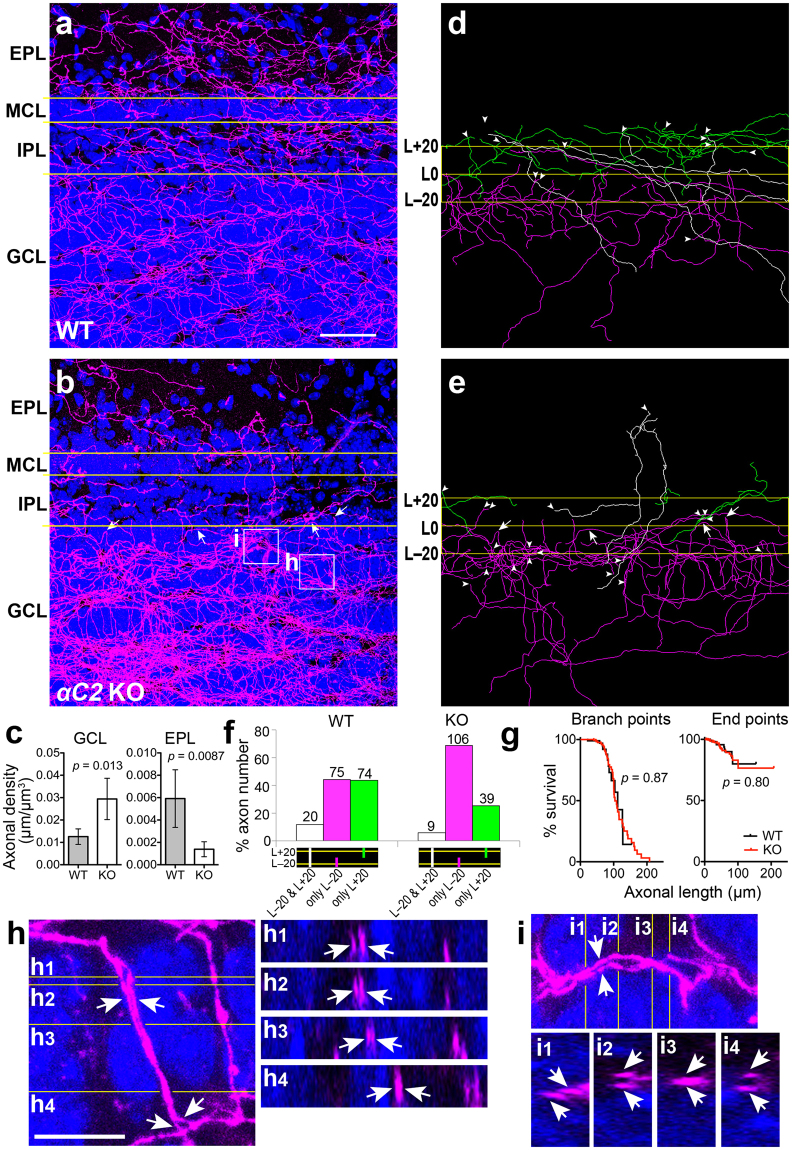
Serotonergic axons in the granule cell layer are suppressed from invading the outer layers in *αC2* KO mice. (**a–c**) In the olfactory bulb, serotonergic axons of *αC2* KO mice (**a**) were densified in the granule cell layer (GCL), and sparsified in the internal plexiform layer (IPL), mitral cell layer (MCL), and external plexiform layer (EPL), compared with WT mice (**b**). Sagittal sections of the dorsal olfactory bulb were stained by an anti-SERT antibody (magenta) and DAPI (blue). In the GCL, the density of SERT(+) axons of *αC2* KO mice (n = 5 sections from 3 mice) was significantly higher than that in WT mice (n = 4 sections, 3 mice) (**c**). In the EPL, the density of SERT(+) axons of *αC2* KO mice (n = 6 sections, 3 mice) was significantly lower than that in WT mice (n = 6 sections, 3 mice) (**c**). (**d–f**) SERT(+) axons crossing the lines 20-µm within (L − 20) and/or 20-µm outside (L + 20) from the IPL/GCL boundary (L0) were traced (**d**,**e**). Panels (d) and (e) were depicted from panels (a) and (b), respectively. White, magenta or green lines indicate axons crossing both lines, only L − 20 or only L + 20, respectively (**d**,**e**). Arrowheads indicate end points (**d**,**e**). SERT(+) axons crossing L − 20 and/or L + 20 were counted in WT (n = 3 sections, 3 mice) and *αC2* KO mice (n = 3 sections, 3 mice) (**f**). (**g**) In the GCL, there was no significant difference in the frequency of branch points or of end points between WT and *αC2* KO mice. SERT(+) axons in the GCL (50 × 50 × 50 µm) of WT (n = 130 axons from 4 sections, 3 mice) and *αC2* KO mice (n = 407 axons from 5 sections, 3 mice) were traced, and their length, branch points, and end points were analyzed. Survival analyses (Log-rank tests) for these axons were performed. (**h**,**i**) SERT(+) axons in GCL of *αC2* KO mice showed fasciculation (arrows in **h** and **i**). Panels (h1)–(h4) and panels (i1)–(i4) indicate the cross sections in panel (h) and (i), respectively. Arrows in **h**,**h1**–**h4**,**i**, and **i1**–**i4** indicate axon pairs that run parallel to each other. Scale bars, 40 µm (**a**,**b**,**d**,**e**); 10 µm (**h–i**).

Layers in the olfactory bulb are separately innervated by median and dorsal raphe nuclei: serotonergic neurons in the median raphe nucleus innervate the glomerular layer (GL), whereas those in the dorsal raphe nucleus innervate the granule cell layer (GCL) to the external plexiform layer (EPL)^17^. Here we focused on the median raphe-innervating layers. SERT(+) axons in *αC2* KO mice were densified in the GCL, and sparsified in the EPL, compared to controls (Fig. 7a–c). To analyze these axonal routes, we traced the SERT(+) axons passing through the 20-µm inner (L − 20) line or 20-µm outer (L + 20) line from the boundary between the GCL and the inner plexiform layer (IPL) (Fig. 7d,e). The probability that SERT(+) axons passing through L − 20 also passed through L + 20 in the *αC2* KO mice was significantly lower than in WT mice [WT, 20 of 95 axons (3 sections from 3 mice); KO, 9 of 115 axons (3 sections from 3 mice), Fisher’s exact test, *p* = 0.0083, Fig. 7f]. In contrast, there was no significant difference in the probability that SERT(+) axons passing through L + 20 also passed through L − 20 between these genotypes (WT, 20 of 94 axons; KO, 9 of 48 axons, Fisher’s exact test, *p* = 0.83, Fig. 7f). Although we cannot determine the axonal direction (outward or inward) from these results alone, these observations raised two possibilities: either the diffusion of outward axons from the GCL to IPL is suppressed, or the arborization of inward axons from the IPL to GCL is facilitated, in the GCL. Therefore, we analyzed SERT(+) axons in the GCL. In this region, there was no significant difference in the occurrence frequency of end points and branch points of SERT(+) axons between WT and *αC2* KO mice (Fig. 7g). These results suggested that the outward diffusion of serotonergic axons from the GCL is suppressed.

The somata of granule cells in the GCL were densely clustered in both genotypes (Fig. 7a,c). Serotonergic axons ran through this cell-cluster niche in a manner avoiding the high-density cell clusters. In the GCL soma niche of *αC2* KO mice, we frequently found serotonergic axons that ran parallel and partially in contact with each other (Fig. 7h,i). We did not detect this phenotype in WT mice. These results suggest that the loss of αC2 protein reduces the axon-axon repulsion of serotonergic axons in the olfactory bulb. As a result, the reduced axonal repulsion may dampen the outward diffusion of serotonergic axons. Around the GCL/IPL boundary, some SERT(+) axons returned to the GCL without contacting other SERT(+) axons (arrows in Fig. 7b,e). This observation suggests that loss of αC2 protein enhances the attraction of serotonergic axons to the GCL or their repulsion from the IPL. The αC2 protein may mediate not only the trans-homophilic interaction-driven repulsion of serotonergic axons, but also the trans-heterophilic interaction-driven axon guidance in target areas.

## Discussion

Here we found that, among the *Pcdh-α* genes, *αC2* was dominantly expressed in the serotonergic neurons of the raphe nuclei, and that the loss of only αC2, and not of α2 to α11, caused unbalanced distributions (densification and sparsification) of serotonergic axon density in various brain regions, including the hippocampus. The abnormalities of the serotonergic projections in *Pcdha* ^*del*(*11-C2*)*/del*(*11-C2*)^ and *Pcdha* ^*∆C2/∆C2*^ mice were very similar to those in *Pcdha* ^*∆CR/∆CR*^ mice^8^. Thus, the diversity of the *Pcdh-α* genes is not required for the normal distribution of serotonergic axons. We also analyzed dorsal telencephalon-specific *Pcdh-α* KO mice and serotonergic neuron-specific *αC2* KO mice. The former showed a normal distribution of serotonergic projections, whereas the latter showed unbalanced distributions similar to those of the *αC2* KO mice. Analyses of serotonergic projections at the single-axon level revealed that the serotonergic-axon diffusion in *αC2* KO mice is suppressed. These results indicated that the *αC2* expression in serotonergic neurons is essential for the normal diffuse pattern of serotonergic axons in various target regions.

Recently, Chen *et al*. also analyzed Pcdh-α function in serotonergic system^18^. (1) They analyzed the expression of *Pcdh-α*, *-β*, and *-γ* by single-cell RNA-seq in serotonergic neurons, and showed that αC2 is intensely expressed in serotonergic neurons. (2) They showed that loss of αC1 and αC2 causes clumping of serotonergic axons in the hippocampus. (3) They showed that loss of Pcdhα in serotonergic neurons causes clumping of serotonergic axons in the hippocampus although loss of Pcdhα in the hippocampal neurons do not. (4) They individually labeled serotonergic axons in serotonergic neuron-specific Pcdh-α KO mice, and found clumped axons in the hippocampus and substantia innominata. From these results, they concluded that αC2 is required for axonal tiling and assembly of serotonergic axons.

Similar abnormal axonal clumps appear in Ia afferent axons in the spinal cord of *Pcdh-γ* KO mice^19^, and in triple KO mutants in *γC3* to *γC5* ^20^. Interestingly, *γC3* to *γC5* are constitutively expressed genes similar to *αC2*. However, analyses of conditional *Pcdh-γ* KO mice revealed that Pcdh-γ expressed in both Ia afferents and their targets (interneurons in the spinal cord) is required to prevent the clumping of projections during the formation of Ia afferent terminals^19^. In the serotonergic projection, *αC2* was required not in the target neurons, but in the serotonergic neurons themselves. Therefore, the mechanism underlying the inhibition of axonal densification in serotonergic axons by αC2 is distinct from that in Ia afferents by Pcdh-γC3–C5.

We previously reported that the cytoplasmic tail of Pcdh-α is essential for normal serotonergic projection^8^. The cytoplasmic domain of αC2 is known to bind cytoplasmic signaling proteins of the focal adhesion kinase (FAK) family, FAK and PYK2^21^. The FAK family contributes to signaling cascades that regulate the growth cone motility, and is activated by several axon-guidance cues, including brain-derived neurotrophic factor (BDNF)^22,23^. Interestingly, BDNF can induce serotonergic axonal growth in the brain^24,25^. Stathmin family proteins, microtubule-destabilizing proteins, also bind to the cytoplasmic domain of Pcdh-α^26^. These proteins are involved in axonal elongation and branching^27^, and their phosphorylation is induced by BDNF^28,29^. Therefore, BDNF and stathmin family proteins are candidate molecules involved in the αC2 signaling cascade that controls serotonergic projections.

Glia cell line-derived neurotrophic factor (GDNF) enhances the neurite elongation of serotonergic neurons *in vitro* ^30^. GDNF family receptor, which is expressed in serotonergic neurons^31^, forms a receptor complex with Ret tyrosine kinase, an essential molecule for GDNF signaling. Ret directly binds to α4, and enhances the phosphorylation of α4′s cytoplasmic domain, in a GDNF dosage-dependent manner^26^. This binding stabilizes both Ret and α4. In serotonergic neurons, the *α4* expression level is considerably lower than that of *αC2*. Nevertheless, GDNF and Ret are also candidates for regulating αC2’s function in the serotonergic projections.

A similar situation is seen for the Down syndrome cell adhesion molecule (DSCAM), which belongs to the immunoglobulin superfamily. Two closely related proteins (Dscam and Dscaml1) are expressed in vertebrates, and 19,008 proteins are generated by distinct combinations of alternative splicings of their homolog in *Drosophila* ^32^. Each DSCAM homophilically mediates negative cell-cell interactions^33^, and these homophilic interactions are responsible for dendrite and axonal self-avoidance. In mice, Dscam is expressed in most retinal ganglion cells and prevents the fasciculation of their dendrites by self-avoidance. Similarly, Dscaml1 is expressed in rod bipolar cell dendrites and prevents the fasciculation and clumping of their dendrites and cell bodies by self-avoidance^34^. Thus, homophilic negative interactions between molecules of a single isoform among many diverse Dscams are sufficient to induce repulsion and prevent fasciculation in homotypic dendrites and axons. The diversity of the *Pcdh-α* genes is not required for the normal diffuse innervation of serotonergic axons. Therefore, our study could not reveal the role of the diversity of clustered *Pcdh-α* genes, but can support the possibility that the diversity of Pcdh-α isoforms mediates the axon and/or dendrite repulsion of cortical and hippocampal neurons, and of Purkinje cells of the cerebellum, which show diverse expression patterns^14,35^, like the *Drosophila* DSCAMs.

Azmitia and Segal analyzed serotonergic axonal routes in detail^36^. From the observation, they speculated that serotonergic projection is regulated by epiphytic guidance, that is, they rely on another group of fibers for their structural support. We found many serotonergic axons crossing GCL/IPL boundary in the olfactory bulb. Because granule cells in GCL project their dendrites to EPL, their may guide serotonergic axons. We found serotonergic axons returning to GCL from GCL/IPL boundary without entering IPL in *αC2* KO mice (arrows in Fig. 7b,e). These results raise a possibility that αC2 in serotonergic axons is required to attach the other fibers and to find their appropriate routes and final target areas.

In this study, we reported that αC2 in serotonergic neurons enables serotonergic axons to extend diffuse projections in various brain areas. Understanding the mechanism by which αC2 promotes diffuse projections in the appropriate brain target regions should shed light on the cellular and molecular mechanisms important for homotypic axonal repulsion and serotonin-related developmental disorders.

## Methods

### Animal experiments

All of the experimental procedures were in accordance with the Guide for the Care and Use of Laboratory Animals of the Science Council of Japan and were approved by the Animal Experiment Committee of Osaka University or the National Institute of Genetics. Male or female mice in adulthood (>3 months old) were used in the axon-tracing analyses. In the other experiments, male or female mice at postnatal day 21 were used.

### Generation of Pcdh-*α* mutant mice

Mice possessing the *Pcdha* ^*del*(*2-11*)^, *Pcdha* ^*del*(*2-11*)^, or *Pcdha* ^*flox*^ alleles were described previously^14,37,38^. To generate dorsal telencephalon-specific *Pcdh-α* KO mice, we crossed *Pcdha flox* mice with *Emx1-Cre KI-∆Neo* mice^15^. To generate serotonergic neuron-specific *Pcdh-α* KO mice, we crossed *Pcdha flox* mice with *ePet-Cre* mice^1^.

To remove the initiation codon of αC2, we generated the SBC2 targeting vector by inserting a PCR-amplified 5′ homologous arm of a 317-base region downstream of the αC2 initiation codon (5.5 kb, from 5′-catactacctgattgtccatc-3′ to 5′-gctgcctctgccagggcc-3′), a sleeping beauty cassette (2.5 kb, *SalI-loxP–IR/DR-L–loxP–neomycin-resistance* (*neor*)*–loxP–IR/DR-R-SalI*)^39^, and a PCR-amplified 5′ homologous arm of a 7-base region upstream of the αC2 initiation codon (5.5 kb, from 5′-cgaggcgctgtgcgagcag-3′ to 5′-ggtgtctttaaaactttaaatgat-3′), into *pMC1DT-A* ^40^ (Supplemental Fig. 1a,b). The linearized vector was introduced into TT2 ES cells by electroporation^41^. Using standard methods, we obtained targeted recombinants and their chimeric offspring. We confirmed them by Southern blot analysis with a 5′ probe (synthesized by the primer set, 5′-tacacactgaaagttaaggc-3′ and 5′-tgtcactccatctactcc-3′), and 3′ probe (synthesized by the primer set, 5′-gaattagtggttagaacctcaccc-3′ and 5′-taggaccaacctaaccacaagaccc-3′) after *Nhe*I and *ApaL*I digestion, respectively (Supplemental Fig. 1c,d). To remove the *neor* sequence from the *SBC2* allele, we crossed the *SBC2* mice with *Sycp-Cre* mice^14^, and obtained mice carrying the *SBC2∆Neo* allele (Supplemental Fig. 1a). To completely remove the coding region of the *αC2* exon from the *SBC2∆Neo* allele by Cre-mediated trans-allelic recombination, we crossed female mice (C57BL/6 J) with male mice carrying the *Sycp-Cre* transgene, *SBC2∆Neo* allele, and *G1* allele, in which the *loxP* sequence was inserted between the *αC2* exon and *CR1* exon^42^, and obtained mice carrying the *∆C2* allele (Supplemental Fig. 1a). *Pcdha* ^+*/∆C2*^ and *Pcdha* ^*∆C2/∆C2*^ mice were fertile. Their genotypes were confirmed by PCR using tail chip DNA. The bands of the WT (967 bp) and ∆C2 alleles (424 bp) were obtained using primers (PcdhaC2(−120):F, 5′-gtagtgcgtgagaggtgaag-3′; PcdhaC2 (+847):R 5′-ggtccgaggcattcaacttc-3′; and G1-GTP-R1, 5′-gcccaggatggctcaaattc-3′) (Supplemental Fig. 1e).

### *In situ* hybridization, histochemistry, and quantitative RT-PCR

*In situ* hybridization, histochemistry, and quantitative RT-PCR were performed by previously described methods^8,14^. Quantitative RT-PCR for the *Sert* transcript used the primer pair: 5′-catcagccctctgtttctcc-3′ and 5′-cttaagtgtccctggagtgc-3′. Control plasmid containing the *Sert* gene was constructed by inserting a PCR amplicon (5′-ctgtcattggctatgccgtg-3′ and 5′-cttaagtgtccctggagtgc-3′) into a *pCR4-Blunt-TOPO* vector (Thermo Fisher Scientific).

### Analysis of serotonergic fiber density

The distribution of serotonergic axons using an anti-serotonin transporter (SERT) antibody (HTT-N77, a generous gift from Dr. Masahiko Watanabe, Hokkaido University, Japan), and the SERT-immunopositive fiber density in the hippocampus were analyzed as described previously with small modifications^8^. All photographs were taken with a DP-72 CCD camera (Olympus) mounted on a BX51 microscope (Olympus). The resolution of the photographs used to analyze serotonergic axon density was 1101 pixel/mm (Fig. 1e,f), or 1643 pixel/mm (Figs 3e, 4f and 5h). Prism software (GraphPad Software, Inc.) was used for the statistical analyses. Statistical comparisons among three mouse lines were done with one-factor ANOVA and Bonferroni’s post hoc test. Statistical comparisons between two mouse lines were done with the Mann-Whitney *U* test. The criterion for significance was p < 0.05.

### Analyses of serotonergic axons at the single-axon level

To label serotonergic neurons that project to the hippocampus, we injected the adeno-associated virus vector pK207_*AAV-EF1α-DIO-tRFP-WPRE* into the median raphe nucleus (angle: 25°, AP: −4.7 mm, ML: 0.2~0.6 mm, DC 5.0 mm from bregma) of mice that were anesthetized by a Univentor 410 Anesthetisia Unit, using a stereotaxic apparatus (Narishige). *5HTT-Cre* (*Sert-Cre*) mice^16^ and *Sert-Cre;Pcdha* ^*∆C2/∆C2*^ mice were used as control and experimental groups, respectively. pK207 was constructed by inserting tRFP (*pTurboRFP-N*, evrogen) into the *Asc*I/*Nhe*I site of pK168_*AAV-EF1α-DIO-tTA-tRFP-WPRE* (Adgene 85039)^43^. Virus was prepared as described previously^43^. More than 4 weeks after the virus injection, mice were transcardially perfused with 4% paraformaldehyde/0.1 M phosphate buffer (PB). The brains were removed and immersed in 30% sucrose/0.1 M PB, and 100-μm-thick sagittal sections in the hippocampus and 100-μm-thick coronal sections in the raphe nuclei were prepared with a microtome (Yamato Kohki). The sections were incubated in rabbit anti-SERT (1:1000, Frontier Institute) [and mouse anti-Cre antibodies (1:1000, Millipore) in some cases] in phosphate buffed saline (PBS) containing 1.5% normal goat serum and 0.25% Triton X-100 for >2 days. After washing with PBS, the sections were incubated in Alexa Fluor 488 anti-rabbit IgG (1:1000) [and Alexa Fluor 647 anti-mouse IgG antibody in some cases] and 4′,6-diamidino-2-phenylindole (DAPI, 1 μg/mg) for >2 days, and washed with PBS. From the sections, z-stack images were captured with a confocal microscope (Leica). In the images, the SLM/SR boundary (L0) was determined by the density difference in DAPI(+) nuclei (high in SLM, low in SR), and lines 20 μm away from the SLM/SR boundary were drawn on both the inner (L − 20) and outer (L + 20) sides. Z-stack images were smoothed by the 3D Gaussian lowpass filter function of ImageJ. All RFP(+) axons crossing both lines in one set of z-stack images (depth: 100 μm) were traced with the ImageJ plugin software, Simple Neurite Tracer^44^. For the statistical analysis, Fisher’s exact test was used.

Sagittal sections of the olfactory bulb were also stained with the anti-SERT antibody and DAPI with the same method. From these sections, z-stack images were captured with the confocal microscope. In the images, the GCL/IPL boundary was determined by the density difference of DAPI(+) nuclei (high in GCL, low in IPL), and lines 20 μm apart from the GCL/IPL boundary were drawn on both the inner (L − 20) and outer (L + 20) sides. All SERT(+) axons crossing both lines in one set of z-stack images (depth: 50 μm) were traced with Simple Neurite Tracer. The length of SERT(+) axons in boxes (50 × 50 × 50 μm) in the GCL and EPL was traced and measured with Simple Neurite Tracer. For the statistical analysis, Welch’s *t*-test was used. In the analyses of branch and end points in boxes (50 × 50 × 50 μm) in the GCL, an axon connecting with branch points was regarded as one axon, and its length was defined as its sum length. In the survival analyses of branch (or end) points, axons with branch (or end) points were dealt with as an event (death), and the other axons were regarded as being censored. For the statistical analyses, the log-rank test was used.

## Electronic supplementary material

Supplementary Information for “Protocadherin-αC2 is required for diffuse projections of serotonergic axons”

## References

[1] Scott MM (2005). A genetic approach to access serotonin neurons for *in vivo* and *in vitro* studies. Proc Natl Acad Sci USA.

[2] Deneris ES (2011). Molecular genetics of mouse serotonin neurons across the lifespan. Neuroscience.

[3] Kiyasova V, Gaspar P (2011). Development of raphe serotonin neurons from specification to guidance. Eur J Neurosci.

[4] Gaspar P, Cases O, Maroteaux L (2003). The developmental role of serotonin: news from mouse molecular genetics. Nat Rev Neurosci.

[5] Lidov HG, Molliver ME (1982). An immunohistochemical study of serotonin neuron development in the rat: ascending pathways and terminal fields. Brain research bulletin.

[6] Donovan, S. L., Mamounas, L. A., Andrews, A. M., Blue, M. E. & McCasland, J. S. GAP-43 is critical for normal development of the serotonergic innervation in forebrain. *J Neurosci* **22**, 3543–3552, doi:20026295 (2002). 10.1523/JNEUROSCI.22-09-03543.2002 PMC6758352 11978831

[7] Fournet V (2010). The deletion of the microtubule-associated STOP protein affects the serotonergic mouse brain network. J Neurochem.

[8] Katori S (2009). Protocadherin-alpha family is required for serotonergic projections to appropriately innervate target brain areas. J Neurosci.

[9] Kohmura N (1998). Diversity revealed by a novel family of cadherins expressed in neurons at a synaptic complex. Neuron.

[10] Wu Q, Maniatis T (1999). A striking organization of a large family of human neural cadherin-like cell adhesion genes. Cell.

[11] Fukuda E (2008). Down-regulation of protocadherin-alpha A isoforms in mice changes contextual fear conditioning and spatial working memory. Eur J Neurosci.

[12] Thu CA (2014). Single-cell identity generated by combinatorial homophilic interactions between alpha, beta, and gamma protocadherins. Cell.

[13] Lefebvre JL, Kostadinov D, Chen WV, Maniatis T, Sanes JR (2012). Protocadherins mediate dendritic self-avoidance in the mammalian nervous system. Nature.

[14] Noguchi Y (2009). Total expression and dual gene-regulatory mechanisms maintained in deletions and duplications of the Pcdha cluster. J Biol Chem.

[15] Iwasato T (2000). Cortex-restricted disruption of NMDAR1 impairs neuronal patterns in the barrel cortex. Nature.

[16] Arakawa H (2014). Thalamic NMDA receptor function is necessary for patterning of the thalamocortical somatosensory map and for sensorimotor behaviors. J Neurosci.

[17] Steinfeld R, Herb JT, Sprengel R, Schaefer AT, Fukunaga I (2015). Divergent innervation of the olfactory bulb by distinct raphe nuclei. J Comp Neurol.

[18] Chen WV (2017). Pcdhalphac2 is required for axonal tiling and assembly of serotonergic circuitries in mice. Science.

[19] Prasad T, Weiner JA (2011). Direct and Indirect Regulation of Spinal Cord Ia Afferent Terminal Formation by the gamma-Protocadherins. Front Mol Neurosci.

[20] Chen WV (2012). Functional significance of isoform diversification in the protocadherin gamma gene cluster. Neuron.

[21] Chen J (2009). alpha- and gamma-Protocadherins negatively regulate PYK2. J Biol Chem.

[22] Woo S, Rowan DJ, Gomez TM (2009). Retinotopic mapping requires focal adhesion kinase-mediated regulation of growth cone adhesion. J Neurosci.

[23] Myers JP, Robles E, Ducharme-Smith A, Gomez TM (2012). Focal adhesion kinase modulates Cdc42 activity downstream of positive and negative axon guidance cues. J Cell Sci.

[24] Mamounas LA, Blue ME, Siuciak JA, Altar CA (1995). Brain-derived neurotrophic factor promotes the survival and sprouting of serotonergic axons in rat brain. J Neurosci.

[25] Mamounas LA (2000). BDNF promotes the regenerative sprouting, but not survival, of injured serotonergic axons in the adult rat brain. J Neurosci.

[26] Schalm SS, Ballif BA, Buchanan SM, Phillips GR, Maniatis T (2010). Phosphorylation of protocadherin proteins by the receptor tyrosine kinase Ret. Proc Natl Acad Sci USA.

[27] Poulain FE, Sobel A (2010). The microtubule network and neuronal morphogenesis: Dynamic and coordinated orchestration through multiple players. Mol Cell Neurosci.

[28] Cardinaux JR, Magistretti PJ, Martin JL (1997). Brain-derived neurotrophic factor stimulates phosphorylation of stathmin in cortical neurons. Brain Res Mol Brain Res.

[29] Jeanneteau F, Deinhardt K, Miyoshi G, Bennett AM, Chao MV (2010). The MAP kinase phosphatase MKP-1 regulates BDNF-induced axon branching. Nat Neurosci.

[30] Ducray A (2006). GDNF family ligands display distinct action profiles on cultured GABAergic and serotonergic neurons of rat ventral mesencephalon. Brain Res.

[31] Sarabi, A., Hoffer, B. J., Olson, L. & Morales, M. Glial cell line neurotrophic factor-family receptor alpha-1 is present in central neurons with distinct phenotypes. *Neuroscienc*e **116**, 261–273, doi:S0306452202005596 [pii] (2003). 10.1016/s0306-4522(02)00559-6 12535958

[32] Zipursky SL, Sanes JR (2010). Chemoaffinity revisited: dscams, protocadherins, and neural circuit assembly. Cell.

[33] Wojtowicz WM (2007). A vast repertoire of Dscam binding specificities arises from modular interactions of variable Ig domains. Cell.

[34] Fuerst PG (2009). DSCAM and DSCAML1 function in self-avoidance in multiple cell types in the developing mouse retina. Neuron.

[35] Esumi S (2005). Monoallelic yet combinatorial expression of variable exons of the protocadherin-alpha gene cluster in single neurons. Nat Genet.

[36] Azmitia EC, Segal M (1978). An autoradiographic analysis of the differential ascending projections of the dorsal and median raphe nuclei in the rat. J Comp Neurol.

[37] Hasegawa S (2016). Distinct and Cooperative Functions for the Protocadherin-alpha, -beta and -gamma Clusters in Neuronal Survival and Axon Targeting. Front Mol Neurosci.

[38] Tarusawa E (2016). Establishment of high reciprocal connectivity between clonal cortical neurons is regulated by the Dnmt3b DNA methyltransferase and clustered protocadherins. BMC Biol.

[39] Takeda J, Keng VW, Horie K (2007). Germline mutagenesis mediated by Sleeping Beauty transposon system in mice. Genome Biol.

[40] Yagi T (1993). A novel negative selection for homologous recombinants using diphtheria toxin A fragment gene. Anal Biochem.

[41] Yagi T (1993). A role for Fyn tyrosine kinase in the suckling behaviour of neonatal mice. Nature.

[42] Hasegawa S (2008). The protocadherin-alpha family is involved in axonal coalescence of olfactory sensory neurons into glomeruli of the olfactory bulb in mouse. Mol Cell Neurosci.

[43] Luo W (2016). Supernova: A Versatile Vector System for Single-Cell Labeling and Gene Function Studies *in vivo*. Sci Rep.

[44] Longair MH, Baker DA, Armstrong JD (2011). Simple Neurite Tracer: open source software for reconstruction, visualization and analysis of neuronal processes. Bioinformatics.

