# Supplementary material for: Protocadherin-αC2 is required for diffuse projections of serotonergic axons
Source: Sci Rep. 2017 Nov 21;7:15908. doi: 10.1038/s41598-017-16120-y (PMC5698425; doi:10.1038/s41598-017-16120-y)
Supplement: Supplementary file 1 — Supplementary Information for “Protocadherin-αC2 is required for diffuse projections of serotonergic axons” [file 41598_2017_16120_MOESM1_ESM.doc]

**Supplementary Information for “Protocadherin-αC2 is required for diffuse projections of serotonergic axons”**

**Authors:** Shota Katori^1, 2^, Yukiko Noguchi-Katori^1^, Atsushi Okayama^1^, Yoshimi Kawamura^3^, Wenshu Luo^2, 4^, Kenji Sakimura^5^, Takahiro Hirabayashi^1^, Takuji Iwasato^2^, Takeshi Yagi^1, 6^*

**Affiliation:** ^1^KOKORO-Biology, Laboratories for Integrated Biology, Graduate School of Frontier Biosciences, Osaka University, 1-3 Yamadaoka, Suita, Osaka, 565-0871, Japan. ^2^Division of Neurogenetics, National Institute of Genetics, 1111 Yata, Mishima, Shizuoka, 411-8540, Japan, ^3^Department of Physiology, Keio University School of Medicine, 35 Shinanomachi, Sinjuku, Tokyo, 113-0021 Japan, ^4^Department of Genetics, SOKENDAI (The Graduate University for Advanced Studies), Mishima, Shizuoka 411-8540, Japan, ^5^Department of Cellular Neurobiology, Brain Research Institute, Niigata University, 1-757 Asahimachidoori, Chuoku, Niigata, 951-8585, Japan, ^6^AMED-CREST, Japan.

***Correspondence to:** yagi@fbs.osaka-u.ac.jp

**
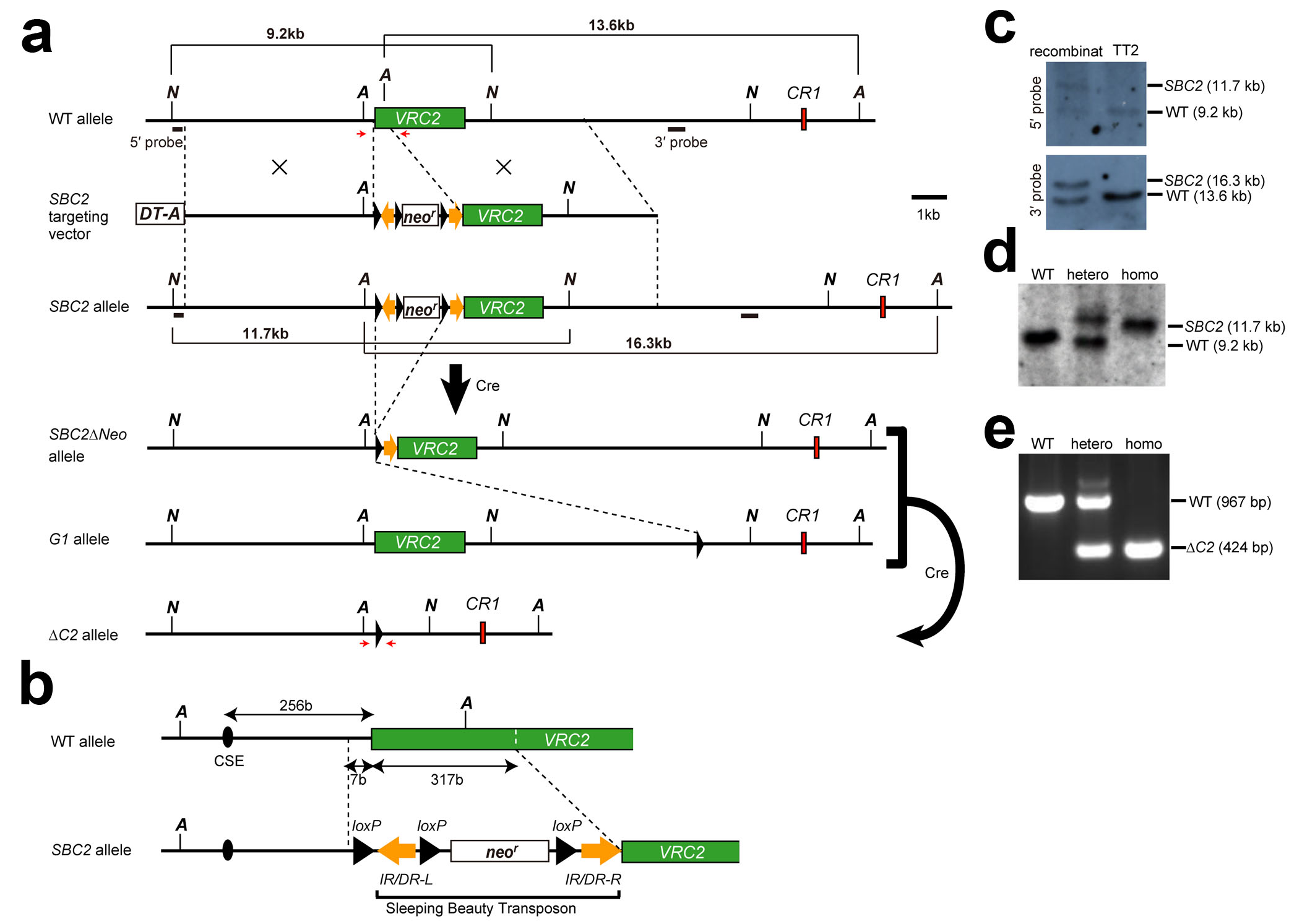
**

**Supplemental figure 1 | Generation of the ∆C2 allele**

a, b) The *SBC2* allele, in which the initiation codon of *PcdhαC2* was removed, was generated by homologous recombination. Homologous regions between the WT allele and *SBC2* targeting vector are delimited by dashed lines. The *SBC2∆Neo* allele was generated by removing *neo^r^* with the Cre-loxP system. The *∆C2* allele, in which the coding region of *VRC2* exon was removed, was generated by Cre-mediated trans-allelic recombination between the *SBC2∆Neo* and *G1* alleles. *N*, *Nhe*I; *A*, *ApaL*I; CSE, conserved sequence element ^43^. c, d) Confirmation of homologous recombination by Southern blotting analysis for targeted ES cells (c) and tail chips of mutant mice carrying the *SBC2* allele (d) with 5´ and 3´ probes. The predicted *Nhe*I- or *ApaL*I-digested DNA fragments are drawn above and below the construct in (a). e) Genotyping PCR of mutant mice carrying the *∆C2* allele. The locations of primers used for the genotyping PCR are indicated in (a) as red arrows.
